# Rethinking sexual response: are erogenous zones—including nipple stimulation—more than secondary stimuli? A meta-analysis

**DOI:** 10.1093/sexmed/qfag065

**Published:** 2026-08-17

**Authors:** Aysu Yildiz Karaahmet, Sevda Karakas, Fatma Sule Bilgic

**Affiliations:** Faculty of Health Sciences, Department of Midwifery, Biruni University, Istanbul, 34100, Turkey; Faculty of Health Sciences, Department of Nursing, Gumushane University, Gumushane, 29100, Turkey; Faculty of Health Sciences, Department of Midwifery, Canakkale Onsekiz Mart University, Canakkale, 17100, Turkey

**Keywords:** nipple stimulation, erogenous zones, sexuality, orgasm

## Abstract

**Objectives:**

The nipple, a key erogenous zone in female sexual response, is often insufficiently considered in clinical practice, and scientific studies on nipple stimulation are limited. The aim of this study was to conduct a systematic review and meta-analysis of studies examining the effect of nipple stimulation on sexual response and sexual satisfaction in young women.

**Methods:**

For this systematic review and meta-analysis, studies were identified through a search of the PubMed, Cochrane Library, EBSCOhost, Embase, Web of Science, PsycINFO, and Scopus databases without any year restrictions.

**Results:**

A total of nine studies (~1276 participants) were included in the systematic review. Of these, six studies (390 intervention, 441 control; n = 831) contributed to the meta-analysis of sexual response/orgasm outcomes, and six studies (655 intervention, 621 control; n = 1276) contributed to the meta-analysis of sexual satisfaction. Nipple stimulation significantly improved sexual response [standardized mean difference (SMD) = 0.51, *P* < .00001] and sexual satisfaction (SMD = 0.59, *P* = .005).

**Discussion:**

Nipple stimulation may be used as a supportive method to enhance sexual function and sexual satisfaction in young women. It may be considered as a complementary approach to sexual counseling and therapy, particularly for women experiencing sexual aversion, difficulty with arousal, and orgasm problems. This meta-analysis suggests that nipple stimulation may have a moderate positive effect on sexual response and sexual satisfaction in young women; however, the findings should be interpreted with caution due to the limited age range of the included population and the heterogeneity across studies.

## Introduction

In female sexual function, erogenous zones are anatomical areas that enhance sexual arousal due to their high concentration of nerve endings and connections to the central nervous system. The nipple, one of the key erogenous zones that enhances sexual arousal in women, plays a significant role in female sexuality from both biological and psychosexual perspectives.^1^ In the female sexual response cycle, stimulation of the breast and nipple enhances sexual arousal and sexual satisfaction through neurophysiological mechanisms. In women, nipple stimulation plays a critical role in the regulation of neurotransmitter release such as oxytocin and dopamine sexual arousal, emotional intimacy, and the orgasmic response.^2^ It has been reported that breast stimulation may have positive effects on sexual arousal, vaginal lubrication, and orgasmic satisfaction by increasing oxytocin release.^3^ Studies in the literature have demonstrated that breast and nipple stimulation in women significantly increases sexual arousal and enhances orgasmic and sexual satisfaction.^3–5^ Nipple stimulation is one of the erogenous stimulations that plays a significant role in the female sexual response. Due to the dense neural network of the breast and nipple, the resulting sensory stimuli can activate the brain’s sexual pleasure center via the central nervous system. In the literature, nipple stimulation is generally evaluated as part of sexual arousal within the context of masturbation or partnered sexual activity; however, the timing of stimulation (eg, during foreplay, during penetration, or both) has not been standardized or is not reported in detail in most studies.^1–6^ Neuroimaging studies on female sexuality indicate that nipple stimulation produces neurophysiological responses similar to clitoral and vaginal arousal, involving activation of the genital sensory cortex.^5^^,^^6^ Furthermore, it is known that nipple stimulation can enhance sexual arousal, emotional intimacy, and the orgasmic response by increasing oxytocin release. For this reason, nipple stimulation is considered an important component in enhancing sexual desire and supporting sexual satisfaction in women.^4–6^ This study was designed to synthesize and critically evaluate the existing literature in order to determine the effects of nipple stimulation on sexual arousal and sexual satisfaction in young women.

### Research aim and questions

The aim of this study was to conduct a systematic review and meta-analysis of studies examining the effect of nipple stimulation on sexual response and sexual satisfaction in young women.

1. What is the current evidence regarding the effect of nipple stimulation on orgasm and sexual response outcomes in young women?
2. What is the current evidence regarding the effect of nipple stimulation on sexual satisfaction in young women?

## Methods

This was a systematic review and meta-analysis. The development of the study protocol and the writing of the report adhered to the PRISMA statement (Preferred Reporting Items for Systematic Reviews and Meta-Analyses). The study protocol was registered with PROSPERO and the registration number CRD420261383471 was obtained on 30 April 2026. The completed PRISMA 2020 checklist and detailed search strategies for all databases are provided in the Supplementary Materials.

### Search strategy

Databases were systematically searched using the following electronic resources up to 30 May 2026: PubMed, Cochrane Library, EBSCOhost, Embase, Web of Science, PsycINFO, and Scopus. In addition, TR Dizin was included to identify potentially relevant studies published in Turkish journals that may not be indexed in major international databases, thereby reducing the risk of language and indexing bias.

All searches were conducted by two independent researchers (A.Y.K. and S.K.), and any potential discrepancies were assessed and resolved by a third researcher. The search strategy was developed based on the PubMed database and adapted for use with the other databases. The term “erogenous zones” was included in the search strategy to maximize search sensitivity; however, only studies specifically addressing nipple stimulation, breast stimulation, or nipple sensitivity were considered eligible for inclusion.

The keywords and Boolean operators used were as follows: (“erogenous zones” OR “nipple stimulation” OR “breast stimulation” OR “nipple sensitivity”) AND (“sexual arousal” OR “sexual response” OR “orgasm” OR “orgasmic function”) AND (“young adults” OR “adults”) The PubMed search was structured as follows: ((“erogenous zones”[All Fields] OR “nipple stimulation”[All Fields] OR “breast stimulation”[All Fields] OR “nipple sensitivity”[All Fields]) AND (“sexual arousal”[All Fields] OR “sexual response”[All Fields] OR “orgasm”[All Fields] OR “orgasmic function”[All Fields]) AND (“young adults”[All Fields] OR “adults”[All Fields])) Additionally, the reference lists of previously published systematic reviews and relevant studies were manually reviewed, and potentially eligible studies were identified and included in the assessment.

### Inclusion criteria

This systematic review and meta-analysis included studies examining the relationship between erogenous zones particularly nipple stimulation and sexual arousal and orgasmic response in young adults. Studies were selected according to the following PICOS criteria:

#### Participants/Population (P)

Samples comprising healthy individuals aged 18–35 years. If the age range was not explicitly stated in the studies, it was deemed sufficient for the sample’s mean age to fall within this range. The study focused on premenopausal women aged 18–35 years. This age range was selected because it generally represents a period of relatively high sexual activity and is not typically affected by menopausal transition-related hormonal changes. Therefore, it provides a relatively stable period in terms of reproductive health and sexual functioning. Accordingly, this age range was selected to obtain a more homogeneous sample with respect to hormonal status and sexual functioning.

#### Exposure/Intervention (I)

Stimulation of erogenous zones, particularly nipple stimulation (nipple stimulation, breast stimulation, nipple sensitivity). Both experimental and observational studies describing these types of stimulation were included.

#### Comparison (C)

No stimulation, placebo conditions, alternative interventions, comparison groups, or studies without an explicit control group, depending on the study design. Comparator conditions varied across studies and included no stimulation, placebo interventions, alternative procedures, comparison groups, or, in some observational studies, the absence of a formal control group.

#### Outcomes (O)

Primary outcomes were defined as sexual arousal and orgasmic response. Within this scope, the level of sexual arousal (eg, subjective arousal scales), physiological arousal measurements, and the frequency or intensity of orgasm were assessed. Secondary outcomes included the duration of the sexual response, arousal threshold, and participants’ subjective responses to arousal.

#### Study design (S)

Randomized controlled trials (RCTs), experimental and pre-post intervention studies, and observational studies (including cross-sectional and clinical observational studies) were included. Studies published in English and Turkish were included in the review without any time restriction.

### Exclusion criteria

Studies of insufficient methodological quality (eg, those lacking appropriate statistical analysis), pilot studies, articles where the full text was unavailable, and studies failing to report necessary data were excluded. Furthermore, studies presenting only a theoretical framework, editorial articles, commentary articles, protocol articles, and systematic reviews were also excluded. Studies using measurement tools whose validity and reliability had not been verified were excluded. Similarly, studies involving only clinical populations (eg, individuals following breast surgery, those with neurological disorders) were also excluded, as these conditions involve clinical factors that could directly influence sexual response. Studies that did not explicitly define erogenous zone stimulation or failed to distinguish nipple stimulation from other types of stimulation were also excluded from the review. Furthermore, studies providing only qualitative data and those lacking a comparison group were not included in the meta- analysis. Finally, studies reporting only non-specific outcomes such as general sexual satisfaction and those that did not directly measure sexual arousal or orgasmic response were also excluded.

### Study selection and data extraction

The studies included in this systematic review were identified from the records obtained during the search process by removing duplicates, followed by reviews of titles, abstracts, and full texts. Study selection was carried out independently by two researchers (A.Y.K. and S.K.); any disagreements that arose were discussed with the involvement of a third researcher and resolved by consensus. During the data extraction process, the data extraction tool developed by the Joanna Briggs Institute was adapted and used in a manner appropriate to the study’s objectives. This approach ensured consistent and systematic data collection across all studies. The following information was extracted from each study: author(s), year of publication, country where the study was conducted, research design, sample size, participants’ age range or mean age, gender distribution, measurement tools used, type of erogenous zone stimulation (particularly nipple stimulation), and outcome variables assessed.

In the assessment of sexual response, both subjective and physiological indicators were considered, depending on the measurement tools used in the studies. In this context, variables such as subjective sexual arousal (eg, self-report arousal scales), orgasm-related outcomes (eg, frequency or intensity of orgasm), and physiological arousal measurements were examined as related but distinct dimensions of sexual response. Studies reporting physiological measurements (eg, genital arousal responses) were also included in the analysis. Due to differences in outcome reporting and the limited number of eligible studies, these dimensions were synthesized under the broader construct of sexual response to enable quantitative comparison across studies; however, findings were interpreted cautiously given the conceptual heterogeneity between outcome types. Erogenous zone stimulation was assessed as defined in the studies, with nipple stimulation classified under the categories of breast stimulation or general erogenous zone stimulation. This classification was undertaken to enhance comparability across different studies. Data extraction was carried out independently by two researchers; the data obtained were then combined into a single dataset and checked by the lead researcher for accuracy and consistency. It was planned to contact the relevant authors regarding studies containing missing or unclear data.

### Assessment of methodological quality

The methodological quality of the included studies was assessed using tools appropriate to their study designs. For randomized controlled trials (RCTs), the updated version of the Cochrane Risk of Bias Tool (ROB-2) was used, while the Risk of Bias in Non-randomized Studies of Interventions (ROBINS-I) tool was used for non-randomized studies.^7^ The ROB-2 tool was used to assess the risk of bias across five key domains: the randomization process, deviations from intervention allocation, missing data, outcome measurement, and selective reporting. The ROBINS-I tool, on the other hand, was applied to cover areas such as confounding variables, participant selection, intervention classification, deviations from the intervention, missing data, outcome measurement and selective reporting. In this study, as observational designs were anticipated to predominate, the control of confounding variables (eg, age, gender, level of sexual experience) and the validity of measurement methods were addressed as critical factors in the methodological quality assessment. The overall quality of the evidence was assessed using the Grading of Recommendations Assessment, Development and Evaluation (GRADE) approach. Within the GRADE system, the quality of evidence for each primary outcome (sexual arousal and orgasmic response) was evaluated according to the domains of risk of bias, inconsistency (heterogeneity), indirectness, imprecision, and publication bias. Methodological heterogeneity between studies, variation in measurement tools, and sample size differences were considered when evaluating the certainty of evidence.

### Statistical analysis

The meta-analysis was conducted using Review Manager (RevMan) 5.4.1 (The Nordic Cochrane Centre, Copenhagen, Denmark) and, where necessary, Comprehensive Meta- Analysis (CMA) software. Heterogeneity between studies was assessed using the chi-square (χ^2^) test and the I^2^ statistic. An I^2^ value in the range of 0%-40% was interpreted as low heterogeneity, 30%-60% as moderate heterogeneity, 50%-90% as high heterogeneity, and 75%-100% as very high heterogeneity (Higgins et al., 2023). Due to expected methodological and measurement differences, the random-effects model was primarily used in the analyses. Sensitivity analyses were conducted using the fixed-effects model in cases where heterogeneity was low. For continuous variables, the standardized mean difference (SMD) was calculated.

where different measurement tools were used across studies, and the mean difference (MD) was calculated where the same measurement tools were used. In studies reporting correlational data, effect sizes were analyzed using Fisher’s z-transformation. A 95% confidence interval (CI) was reported for all analyses, and a *P*-value of <.05 was considered statistically significant in two-tailed tests. Subgroup analyses were conducted, where possible, by gender (female/male) and type of stimulation (nipple stimulation vs. other erogenous zones). Furthermore, sensitivity analyses were performed to assess the robustness of the results.

Subgroup analyses and meta-regression were considered to explore potential sources of heterogeneity where methodologically appropriate. However, due to the limited number of studies available for each outcome and substantial variation in study characteristics and measurement approaches, formal subgroup analyses and meta-regression were not performed, as these methods may produce unstable and potentially misleading estimates when based on a small number of studies.

## Results

**Figure 1:**
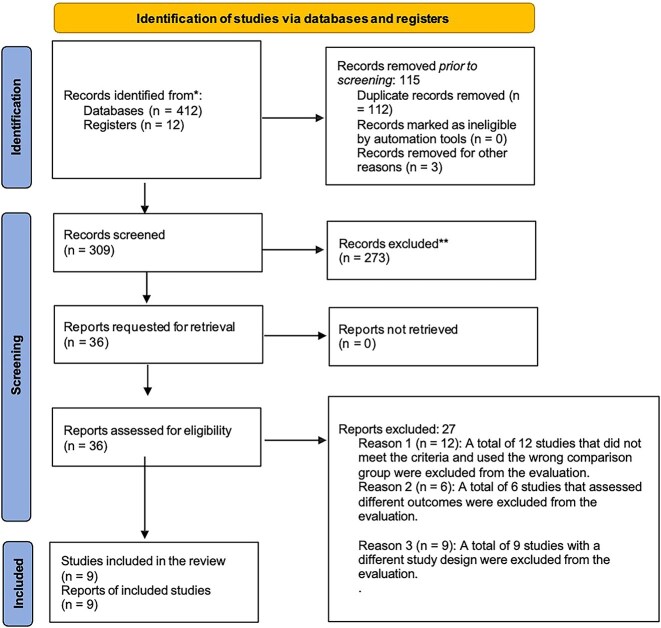
PRISMA flow diagram PRISMA: Preferred reporting items for systematic reviews and meta-analyses.

Figure 1 presents the PRISMA flowchart summarizing the literature review and the study selection process.

### Included studies

In this study, 412 records were identified in the initial database search, and 12 additional records were identified through supplementary searches. After duplicate removal and title/abstract screening, 36 studies were assessed for full-text eligibility. Following evaluation against the inclusion criteria, nine studies were included in the systematic review. However, not all included studies provided sufficient or compatible quantitative data for every outcome; therefore, the number of studies contributing to each meta-analysis varied according to outcome availability. Specifically, six studies contributed to the sexual response/orgasm analysis (n = 831 participants) and six studies contributed to the sexual satisfaction analysis (n = 1276 participants). The reasons for excluding the remaining studies were as follows: 11 studies were neither RCTs nor quasi-experimental studies, two were review studies, nine had inaccessible full texts, three lacked an appropriate comparison/control group, and two reported outcomes incompatible with the predefined meta-analysis outcomes (Figure 1).

### Characteristics of the studies and participants

This systematic review and meta-analysis included nine studies involving a total of ~1276 participants, with the aim of evaluating the effect of nipple stimulation on sexual response. The characteristics of the included studies and participants are summarized in Table 1. The studies were published between 2006 and 2024 and all examined the relationship between nipple or erogenous zone stimulation and sexual response, arousal, or sexual satisfaction.

**Table 1:** Characteristics of the studies included in the systematic review.

**Author, Year**	**Country**	**Study design**	**Sample size**	**Gender**	**Intervention/Exposure**	**Control**	**Measurement tool**	**Outcomes**
Abramsohn *et al.*, 2023	USA	Observational	300	Female	Breast sensory function	None	Breast Sensory Scale	Sexual function, satisfaction
Dossett *et al*., 2020	USA	Observational (clinical)	53	Female	Nipple-sparing mastectomy	SSM	Quality of Life (QOL) scale	Satisfaction
Haider *et al*., 2015	NR	Observational	75	Female	Nipple sensitivity	Comparison groups	FSFI	Orgasm, satisfaction
Harrison *et al*., 2013	USA	Observational	NR	Both	Nipple response (emotional/somatic)	None	Self-report	Indirect arousal
Krychman *et al*., 2019	USA	RCT	59	Female	Topical nipple stimulation	Placebo	FSFI	Orgasm, satisfaction
Levin & Meston, 2006	USA	Cross-sectional	301	Both	Nipple stimulation	None	Self-report	Arousal
Perez *et al*., 2024	NR	Pre-post	50	Female	Erogenous stimulation	None	SDI	Sexual desire
Wise *et al*., 2016	NR	Experimental	11	Female	Imagined nipple stimulation	None	Self-report	Arousal
Younis *et al*., 2016	NR	Observational	130	Female	Extragenital erogenous zones	None	Questionnaire	Sexual pleasure

The data were collected from various countries. All included studies reported outcomes related to sexual response. Primary outcomes include sexual arousal, orgasm, and sexual satisfaction. Secondary outcomes examined include sexual desire, sensory function, and erogenous zone assessments. The most commonly used measurement tools include the Female Sexual Function Index (FSFI) and self-report questionnaires. According to the methodological quality assessment, the overall quality of the included studies ranged from low to moderate- high. The main factors limiting the quality of evidence were identified as the lack of randomization in most studies, the absence of blinding, self-report-based measurements, and high heterogeneity. In particular, observational and experimental designs carry a higher risk of bias due to potential confounding variables. The quality assessments of the included studies are presented in Table 1.

### Primary outcome

#### Meta-analysis findings on the effect of nipple stimulation on sexual response outcomes

**Figure 2:**
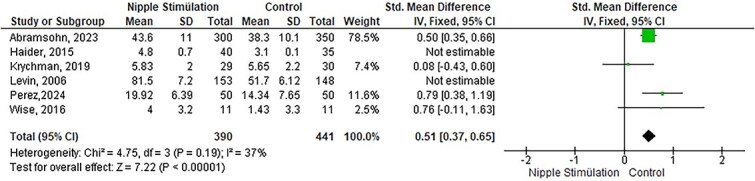
Meta-analysis of the effect of nipple stimulation on orgasm compared to control conditions.

A total of six studies were included in the quantitative synthesis, comprising 390 participants in the nipple stimulation group and 441 in the control group. The pooled analysis demonstrated that nipple stimulation was associated with a statistically significant improvement in overall sexual response outcomes compared to control conditions (SMD = 0.51, 95% CI: 0.37-0.65, *P* < .00001; Figure 2). Heterogeneity across studies was low to moderate (χ^2^ = 4.75, df = 3, *P* = .19; I^2^ = 37%), indicating acceptable consistency among the included studies. The overall effect size was in the moderate range, suggesting that nipple stimulation has a meaningful positive impact on sexual response outcomes.

#### Meta-analysis findings on the effect of nipple stimulation on satisfaction

**Figure 3:**
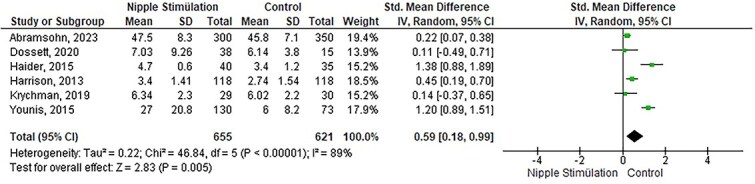
Meta-analysis of the effect of nipple stimulation on sexual satisfaction compared to control conditions.

A total of six studies were included in the meta-analysis of sexual satisfaction, comprising 655 participants in the nipple stimulation group and 621 in the control group. The pooled analysis showed that nipple stimulation was associated with a statistically significant improvement in sexual satisfaction compared to control conditions (SMD = 0.59, 95% CI: 0.18-0.99, *P* = .005; Figure 3). However, substantial heterogeneity was observed across studies (χ^2^ = 46.84, df = 5, *P* < .00001; I^2^ = 89%), indicating considerable variability in the effect estimates. The high heterogeneity suggests that the magnitude of the effect varies significantly across studies likely due to differences in study design, measurement tools, and definitions of sexual satisfaction. Therefore, the pooled estimate for sexual satisfaction should be interpreted cautiously, as the observed heterogeneity may limit the precision and interpretability of the summary effect.

### Risk of bias

The methodological quality of the included studies varied across study designs. One randomized controlled trial was rated as having a low risk of bias. Most observational studies were assessed as having a moderate risk of bias due to reliance on self-reported measures and potential confounding factors. Several studies were rated as having a high risk of bias, particularly those with indirect outcome measures, a lack of control groups, or experimental designs that did not directly assess sexual response. Overall, the evidence base was characterized by a moderate to high risk of bias. Given the moderate to high risk of bias across several included studies, the pooled findings should be interpreted cautiously. In particular, self-reported outcome measures, lack of blinding, and observational designs may have influenced the magnitude and consistency of the observed effects. According to the GRADE framework, the certainty of evidence for the primary outcomes was considered low to moderate, primarily due to methodological limitations, heterogeneity, and imprecision across studies.

Detailed risk-of-bias assessments for all included studies using ROB-2 and ROBINS-I are presented in the Supplementary Materials (Table S1). According to the GRADE framework, the certainty of evidence for the primary outcomes was considered low to moderate, primarily due to methodological limitations, heterogeneity, and imprecision across studies.

## Discussion

The aim of this study was to conduct a systematic review and meta-analysis of studies examining the effect of nipple stimulation on sexual response and sexual satisfaction in young women. The analysis revealed that nipple stimulation may have a moderate positive effect on sexual response and sexual satisfaction. The findings support the notion that the role of nipple stimulation in sexual response may be grounded not only in psychological but also in neurophysiological mechanisms.

Neuroimaging and sexual function studies indicate that genital and non-genital erogenous zones are strongly associated with cortical representation areas, and that activation of the insula, somatosensory cortex, and limbic system plays a central role in sexual arousal processes.^8^ These findings reveal that the sexual response is not solely dependent on genital stimulation, but that different sensory inputs integrate at the level of the central nervous system to shape the sexual experience. In the literature, nipple stimulation is typically examined within masturbation or partnered sexual activity contexts; however, its timing (eg, foreplay or penetration) is rarely standardized or reported in detail in most studies.^8–14^ Indeed, it has been reported that direct stimulation of the somatosensory cortex can elicit genital sensations in some individuals, and that this effect may vary between genders.^10–14^

Neurophysiological evidence regarding the effects of erogenous zone stimulation on the central nervous system supports these findings. In particular, it has been shown that afferent signals from erogenous zones are associated with activation of the insula and somatosensory cortex, and that these processes can enhance sexual arousal^13^ However, it is emphasized that the sexual response is not a unidimensional process, and that subjective arousal, physiological response, and behavioral outcomes do not always progress simultaneously.^15^^,^^16^ This highlights the importance of multidimensional measurement approaches in the assessment of the sexual response. It is increasingly emphasized in the literature that sexual function exhibits not only a neurophysiological but also a biopsychosocial structure. In particular, it has been demonstrated that lifestyle factors, general health status, and psychological variables are decisive for sexual function, and that sexual dysfunction has a multifactorial etiology.^17^ From a clinical perspective, this multidimensional structure must be taken into account when assessing female sexual dysfunction, and not only biological but also psychosocial factors should be included in the analysis.^14–17^ Systematic and meta-analytic studies on the cortical organization of genital sensory representation areas indicate that both men and women possess a distinct and functional representation area for genital regions within the somatosensory cortex.^17–19^ Functional neuroimaging findings reveal that, alongside these cortical areas, the simultaneous activation of the insula and limbic structures plays a role in the processes of sexual arousal and orgasm.^18^ Furthermore, more recent studies indicate that there are structural differences in the female genital representation area related to use and sensory experience, and that this plasticity may influence sexual sensory processing.^20^ Neuroimaging studies have shown that sexual arousal is not limited to genital stimulation but is a complex process involving the integration of multisensory inputs at the level of the central nervous system. In addition, it has been reported that brain activation during sexual arousal may vary according to gender.^19–23^ In this context, it is suggested that non-genital erogenous zones, such as the nipple, may also be involved in sexual response networks.

Experimental studies conducted in recent years suggest that sexual arousal can be modulated using neuromodulation techniques. In particular, a randomized controlled trial reported that repetitive transcranial magnetic stimulation can increase levels of sexual arousal in women.^18^ This finding supports the notion that the sexual response is a process that can be modulated at the level of the central nervous system. Furthermore, it has been shown that masturbation behavior patterns in young adults are associated with sexual arousal and that sexual behavior can influence arousal levels.^19^ This suggests that the sexual response involves not only physiological but also behavioral and learned components. However, it should be noted that the studies included in this meta-analysis primarily assessed behavioral and self-reported sexual outcomes. Therefore, the neuroimaging findings discussed above should be considered as potential explanatory frameworks rather than direct mechanistic evidence for the observed pooled effects.

A substantial degree of heterogeneity was observed in the sexual satisfaction analysis (I^2^ = 89%), which warrants cautious interpretation of the pooled estimate. Several factors may have contributed to this variability, including differences in study design (experimental versus observational), measurement tools used to assess sexual satisfaction, participant characteristics, and variations in the operationalization and context of nipple stimulation across studies. In addition, differences in comparator conditions and outcome definitions may also have influenced the observed effect sizes. Because only a limited number of studies were available for quantitative synthesis, formal subgroup analyses or meta-regression were considered methodologically underpowered and were therefore not performed. Consequently, the pooled estimate for sexual satisfaction should be interpreted cautiously and regarded as exploratory rather than definitive.

This meta-analysis found that nipple stimulation among healthy young women may significantly enhance sexual response. The findings are consistent with the current literature suggesting that stimulation of erogenous zones can modulate the sexual response through central nervous system-based multisensory integration processes. Overall, the current evidence suggests that the sexual response is not solely dependent on genital mechanisms, but rather is shaped by the interaction of neurophysiological, behavioral and psychosocial processes. The findings further indicate a potential association between stimulation of erogenous zones and sexual response; however, causal mechanisms remain to be established.

Furthermore, the methodological quality of the included studies varied, with several studies presenting a moderate to high risk of bias. Therefore, the observed pooled effects should be interpreted cautiously, and future studies employing rigorous randomized designs and standardized outcome measures are required to strengthen the certainty of evidence.

It should also be noted that the construct of sexual response included multiple related but distinct dimensions, including subjective arousal and orgasm-related outcomes. Because these constructs may involve partially different neurophysiological mechanisms, the pooled estimate should be interpreted as reflecting an overall sexual response tendency rather than a single homogeneous outcome.

## Conclusion

This systematic review and meta-analysis demonstrates that erogenous zone stimulation, particularly nipple stimulation, may have a statistically significant and clinically moderate effect on sexual arousal and orgasmic response in healthy young adults. A total of nine studies were included in the systematic review, although the number of studies contributing to each quantitative synthesis varied according to outcome availability. The findings suggest that nipple stimulation may enhance both subjective levels of sexual arousal and the orgasmic response, and may also have positive effects on sexual satisfaction. However, the variability in effect sizes across studies and the high heterogeneity observed, particularly in the analysis of sexual satisfaction, limit the generalizability of the results. In addition, because the review focused on healthy young adults aged 18-35 years, the findings may not be generalizable to older adults or other populations. Although the current evidence provides important insights into the role of erogenous zone stimulation in sexual response, it is not sufficiently robust to allow for definitive conclusions. Therefore, there is a need for future well-designed, randomized controlled trials with adequate sample sizes and the use of standardized measurement tools to clarify this relationship more clearly.

## Supplementary Material

Supplemantary_Search_Strategy_qfag065

Supplemantary_Table_2_qfag065

## Data Availability

The dataset is available from the corresponding author upon reasonable request.
