# Supplementary material for: Rethinking sexual response: are erogenous zones—including nipple stimulation—more than secondary stimuli? A meta-analysis
Source: Sex Med. 2026 Aug 17;14(5):qfag065. doi: 10.1093/sexmed/qfag065 (PMC13480467; doi:10.1093/sexmed/qfag065)
Supplement: Supplementary file 1 [file supplemantary_search_strategy_qfag065.docx]

**Supplementary Table S1. Detailed Search Strategy for All Databases**

### **PubMed**

**Search date:** 30 February 2026

**Filters:** No date restriction applied

(("erogenous zones"[All Fields] OR "nipple stimulation"[All Fields]

OR "breast stimulation"[All Fields] OR "nipple sensitivity"[All Fields])

AND ("sexual arousal"[All Fields] OR "sexual response"[All Fields]

OR "orgasm"[All Fields] OR "orgasmic function"[All Fields])

AND ("young adults"[All Fields] OR "adults"[All Fields]))

### **Cochrane Library**

**Search date:** **:** 30 February 2026

**Filters:** No date restriction applied

("erogenous zones" OR "nipple stimulation" OR "breast stimulation"

OR "nipple sensitivity")

AND

("sexual arousal" OR "sexual response" OR "orgasm"

OR "orgasmic function")

AND

("young adults" OR adults)

### **EBSCOhost**

(Databases searched: MEDLINE, Academic Search Complete, CINAHL)
**Search date:** **:** 30 February 2026

**Filters:** No date restriction applied

TX ("erogenous zones" OR "nipple stimulation"

OR "breast stimulation" OR "nipple sensitivity")

AND

TX ("sexual arousal" OR "sexual response"

OR orgasm OR "orgasmic function")

AND

TX ("young adults" OR adults)

### **Embase**

**Search date:** **:** 30 February 2026

**Filters:** No date restriction applied

('erogenous zone'/exp OR 'nipple stimulation'

OR 'breast stimulation' OR 'nipple sensitivity')

AND

('sexual arousal'/exp OR 'sexual response'

OR orgasm OR 'orgasmic function')

AND

('young adult'/exp OR adult)

### **Web of Science**

**Search date:** **:** 30 February 2026

**Filters:** No date restriction applied

TS=("erogenous zones" OR "nipple stimulation"

OR "breast stimulation" OR "nipple sensitivity")

AND

TS=("sexual arousal" OR "sexual response"

OR orgasm OR "orgasmic function")

AND

TS=("young adults" OR adults)

### **PsycINFO**

**Search date:** **:** 30 February 2026

**Filters:** No date restriction applied

AB ("erogenous zones" OR "nipple stimulation"

OR "breast stimulation" OR "nipple sensitivity")

AND

AB ("sexual arousal" OR "sexual response"

OR orgasm OR "orgasmic function")

AND

AB ("young adults" OR adults)

### **Scopus**

**Search date:** **:** 30 February 2026

**Filters:** No date restriction applied

TITLE-ABS-KEY ("erogenous zones"

OR "nipple stimulation"

OR "breast stimulation"

OR "nipple sensitivity")

AND

TITLE-ABS-KEY ("sexual arousal"

OR "sexual response"

OR orgasm

OR "orgasmic function")

AND

TITLE-ABS-KEY ("young adults" OR adults)

### **TR Dizin**

**Search date:**

**:** 30 February 2026

**Filters:** No date restriction applied

("erojen bölgeler" OR "meme ucu stimülasyonu"

OR "meme stimülasyonu" OR "meme ucu duyarlılığı")

AND

("cinsel uyarılma" OR "cinsel yanıt"

OR orgazm OR "orgazmik işlev")

AND

("genç yetişkinler" OR yetişkinler)
