# Supplementary material for: Rethinking sexual response: are erogenous zones—including nipple stimulation—more than secondary stimuli? A meta-analysis
Source: Sex Med. 2026 Aug 17;14(5):qfag065. doi: 10.1093/sexmed/qfag065 (PMC13480467; doi:10.1093/sexmed/qfag065)
Supplement: Supplementary file 2 [file supplemantary_table_2_qfag065.docx]

**Supplementary Table S2. Detailed Risk of Bias Assessment of Included Studies (ROB-2 and ROBINS-I)**

| **Study** | **Design** | **Assessment Tool** | **Selection Bias** | **Measurement Bias** | **Confounding** | **Overall Risk** |
| --- | --- | --- | --- | --- | --- | --- |
| Abramsohn et al., 2023 | Observational | ROBINS-I | Low | Moderate (self-report outcomes) | Moderate | Moderate |
| Dossett et al., 2020 | Clinical observational (non-randomized) | ROBINS-I | High | Moderate | High | High |
| Haider et al., 2015 | Observational | ROBINS-I | Moderate | Moderate | Moderate | Moderate |
| Harrison et al., 2013 | Observational | ROBINS-I | Moderate | High (indirect outcome measures) | Moderate | High |
| Krychman et al., 2019 | Randomized controlled trial (RCT) | ROB-2 | Low | Low | Low | Low |
| Levin & Meston, 2006 | Cross-sectional | ROBINS-I | Moderate | Moderate (self-report outcomes) | Moderate | Moderate |
| Perez et al., 2024 | Pre-post intervention | ROBINS-I | Moderate | Moderate | High (absence of control group) | High |
| Wise et al., 2016 | Experimental | ROBINS-I | Moderate | High (imagined stimulation/self-report) | Moderate | High |
| Younis et al., 2016 | Observational | ROBINS-I | Moderate | High (subjective erogenous zone assessment) | Moderate | High |

**Abbreviations:** ROB-2 = Revised Cochrane Risk of Bias Tool for Randomized Trials; ROBINS-I = Risk Of Bias In Non-randomized Studies of Interventions.
